# Magnetic Resonance Imaging (MRI) in Parkinson’s Disease

**DOI:** 10.4172/2161-0460.S1-001

**Published:** 2013-03-25

**Authors:** Paul J Tuite, Silvia Mangia, Shalom Michaeli

**Affiliations:** 1 Department of Neurology, University of Minnesota, Minneapolis, MN, USA; 2 Department of Radiology, Center for Magnetic Resonance Research (CMRR), University of Minnesota, Minneapolis, MN, 55455 USA

**Keywords:** Adiabatic methods R_1ρ_ and R_2ρ_, Diffusion Tensor Imaging (DTI), Magnetic Resonance Spectroscopy (MRS), Magnetization transfer imaging (MTI), Relaxations along a Fictitious Field (RAFF), Resting-state MRI, Susceptibility-weighted imaging (SWI), T_2_^*^, Voxel-based morphometry (VBM)

## Abstract

Recent developments in brain imaging methods are on the verge of changing the evaluation of people with Parkinson’s disease (PD). This includes an assortment of techniques ranging from diffusion tensor imaging (DTI) to iron-sensitive methods such as T_2_^*^, as well as adiabatic methods R_1ρ_ and R_2ρ_, resting-state functional MRI, and magnetic resonance spectroscopy (MRS). Using a multi-modality approach that ascertains different aspects of the pathophysiology or pathology of PD, it may be possible to better characterize disease phenotypes as well as provide a surrogate of disease and a potential means to track disease progression.

## Introduction

This brief review focuses on magnetic resonance imaging (MRI) of Parkinson’s disease (PD), which is the second most common neurodegenerative disease after Alzheimer’s disease (AD). Approximately 1% of those over 65 years of age have PD [1]. While MRI is presently not able to directly image dopaminergic neuronal loss that underlies PD, it can provide complementary data to that obtained with nuclear tracer imaging. This article will review commonly available and research MRI methods that may provide an imaging measure of disease.

## T_2_ and T_2_^*^ Imaging

In the 1980s MRI imaging was first applied in PD, when several groups focused on demonstrating the presence of increased iron in the substantia nigra of individuals with PD [2]. This was followed by Gorell et al. in 1995 who utilized T_2_ and T_2_^*^ imaging of the substantia nigra and showed a separation between those with PD from control participants by using a change in relaxation time constants as a surrogate for increased iron in PD [3]. The focus on T_2_^*^or its reciprocal R_2_^*^has remained an important aspect of nigral imaging protocols, and an excellent demonstration of macroscopic nigral changes attributable to iron was shown by Cho et al. in their 7 Tesla (T) imaging study [4]. One crucial assumption about “iron” based imaging is that the methods reflect upon non-heme iron as opposed to heme-iron, and that while the small pool of free labile iron may be pathogenic - imaging methods are presently sensitive to the more prevalent bound iron that is stored as ferritin or neuromelanin [5]. Today MRI cannot determine if these iron changes arise from neuromelanin in dopaminergic neurons or ferritin in glia or neurons [6]. It is thought that the increased stores of bound-iron in PD as compared to iron accumulation with “normal” aging, may represent a source of additional free and pathogenic iron [5]. Other important issues to consider in iron imaging studies are dietary, environmental and gender factors, and some propose using serum ceruloplasmin along with brain imaging to address some of these confounds [7]. Finally, while iron changes are present in PD, validation of iron-based imaging as a surrogate of disease remains to be determined. Meanwhile iron-sensitive methods other than T_2_^*^ have been developed, and remain to be validated and employed on a larger scale, and include adiabatic T_2ρ_ and susceptibility-weighted imaging (SWI) [8–14].

## Susceptibility-weighted Imaging (SWI)

SWI methods exploit the differences in magnetic susceptibility between tissues, and are available on clinical MRI platforms. Using gradient echo (GRE) pulse sequences with long echo time (TE), SWI provides enhanced image contrast for detecting susceptibility variations when combining magnitude and phase data. Specifically, the local field variations are the source of local phase differences in the MRI signal. Phase variations contain both microscopic and macroscopic effects. The phase variations due to microscopic effects mainly originate from local iron deposits whereas the macroscopic effects can be attributed to geometry effects or air/tissue interfaces. Complicated tissue geometries such as capillary beds, interstitial spaces, large and small vessels, etc., distort the local field homogeneity and thus induce signal variations. In fact, induced susceptibility differences depend not only on the geometry of such structures, but also on their orientations with respect to the external magnetic field.

**Figure 1:**
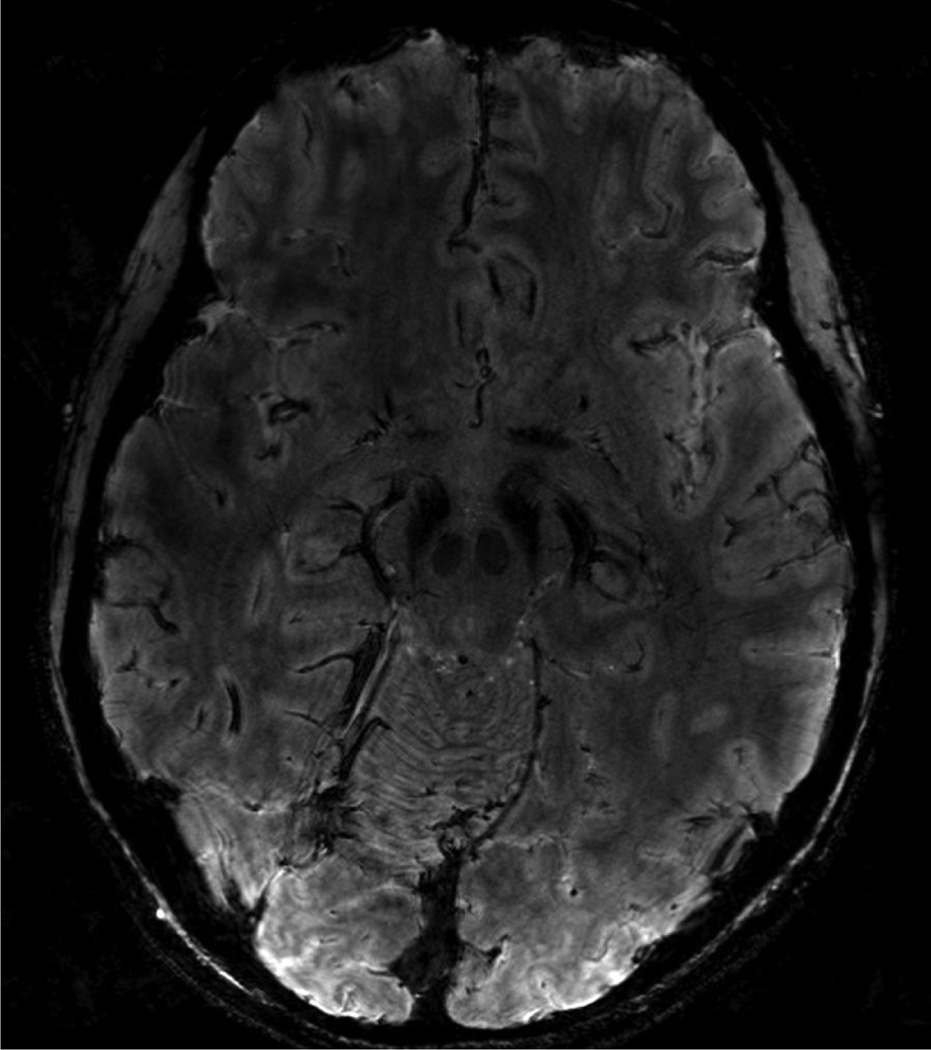
Susceptibility weighted imaging (SWI) acquired at a high-field (7T) MRI. Image resolutions 0.4 × 0.4 × 0.8 mm^3^. The axial image at the levels of the substantia nigra (SN) level. Images courtesy of Dr. Noam Harel, University of Minnesota.

**Figure 2:**
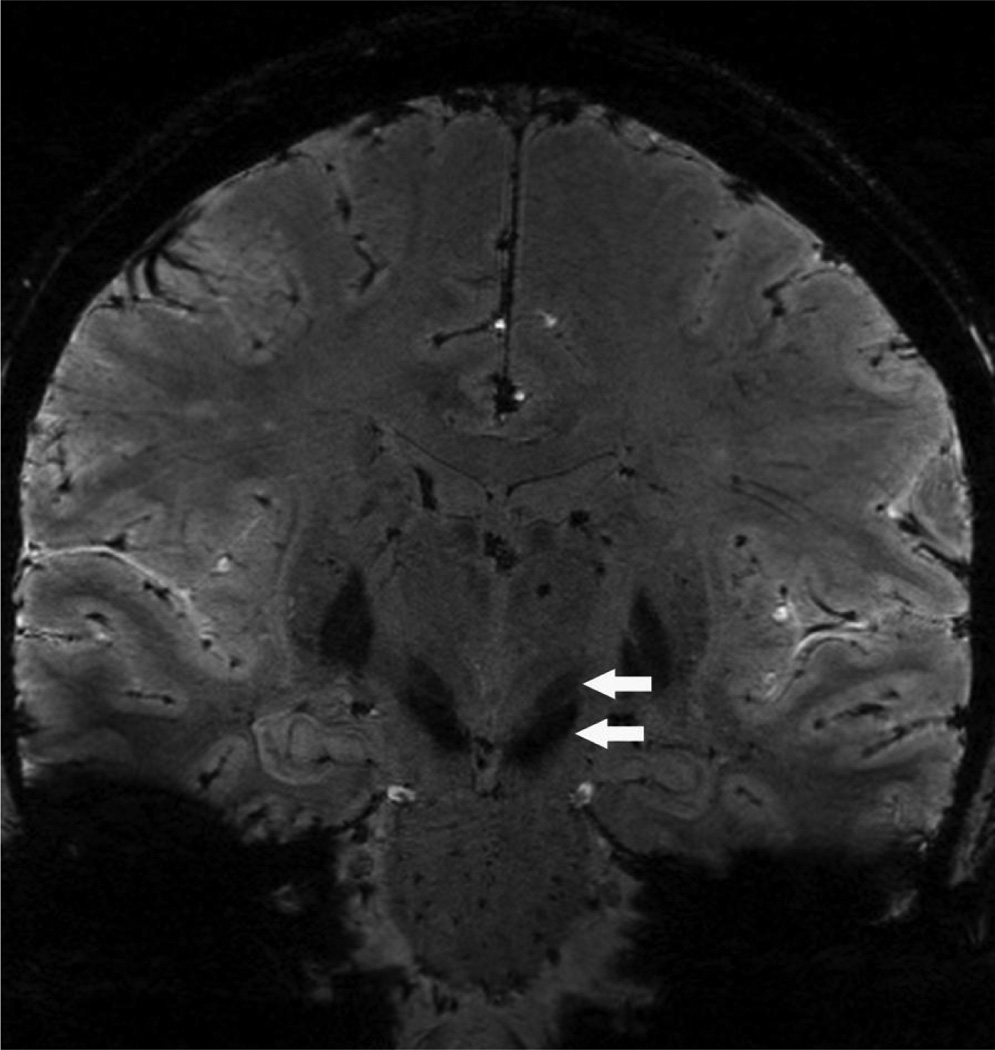
Susceptibility weighted imaging (SWI) acquired at a high-field (7T) MRI. Image resolutions 0.4 × 0.4 × 0.8 mm^3^. Coronal view shows a clear delineation between the subthalamic nucleus (STN) (higher arrow) and the substantia nigra (lower arrow). Images courtesy of Dr. Noam Harel, University of Minnesota.

SWI is also sensitive to the presence of deoxygenated blood, ferritin, calcium, iron as well as transition metals such as Mn^2+^ or Cu^2+^. However, due to the low concentrations of most of the aforementioned substances, disruption of magnetic field homogeneity and the resultant signal loss can be attributed mainly to iron. Accordingly, quantification of SWI measures can be used as a marker for iron content. The information on the presence of iron in tissue has tremendous importance for neurological disorders, especially PD. Notably, SWI has exquisite capability to highlight anatomical structures which contain iron [15]. As depicted in figures 1 and 2, SWI imaging at 7T shows excellent visualization of deep brain stimulation (DBS) surgical targets and thereby may aid in lead placement for patients undergoing DBS surgery [10–14].

## Magnetization Transfer Imaging (MTI)

Magnetization transfer imaging (MTI) utilizes the transfer of magnetization between free water protons and protons associated with macromolecules which provides information about tissue integrity [16]. The detection of the magnetization transfer (MT) effect in clinical practice is usually limited to the measurement of MT ratios (MTRs), i.e. ratios of signal intensity measured with and without the off resonance saturation pulse [16]. One group has shown the utility of MTR in PD while others have shown its value in atypical parkinsonian conditions [17–21]. In contrast to MTR we have developed an easy-to-implement quantitative magnetization transfer (MT) method to estimate magnetization transfer parameters, which relies on an inversion-prepared MT protocol [22]. Using the inversion-prepared MT protocol together with adiabatic T_1ρ_ we evaluated the integrity of the brainstem structures of PD [23]. Results from this study will be discussed in the next section.

## Adiabatic Rotating Frame Relaxation Methods

Conventionally, MRI contrast is generated by the tissue variation of longitudinal (time constant, T_1_) and/or transverse (time constant, T_2_) relaxation of the ^1^H_2_O MR signals. These time constants are measured in the laboratory frame, in which the direction of the main magnetic field defines the longitudinal or Z axis. The free precession relaxation rate constant (R_1_≡1/T_1_) is sensitive to magnetic fluctuations that occur as a result of molecular motion near the Larmor precession frequency (ω_0_) which falls in the MHz range. However, there is reason to also probe lower frequencies of the non-homogeneously broadened line of tissue (i.e., in the kHz range) in order to evaluate for pathology. Rotating frame relaxation rate constants, R_1ρ_ and R_2ρ_characterize relaxation during radiofrequency (RF) irradiation when the magnetization vector is aligned along or perpendicular to the direction of the effective magnetic field (ω_eff_), respectively.

**Figure 3:**
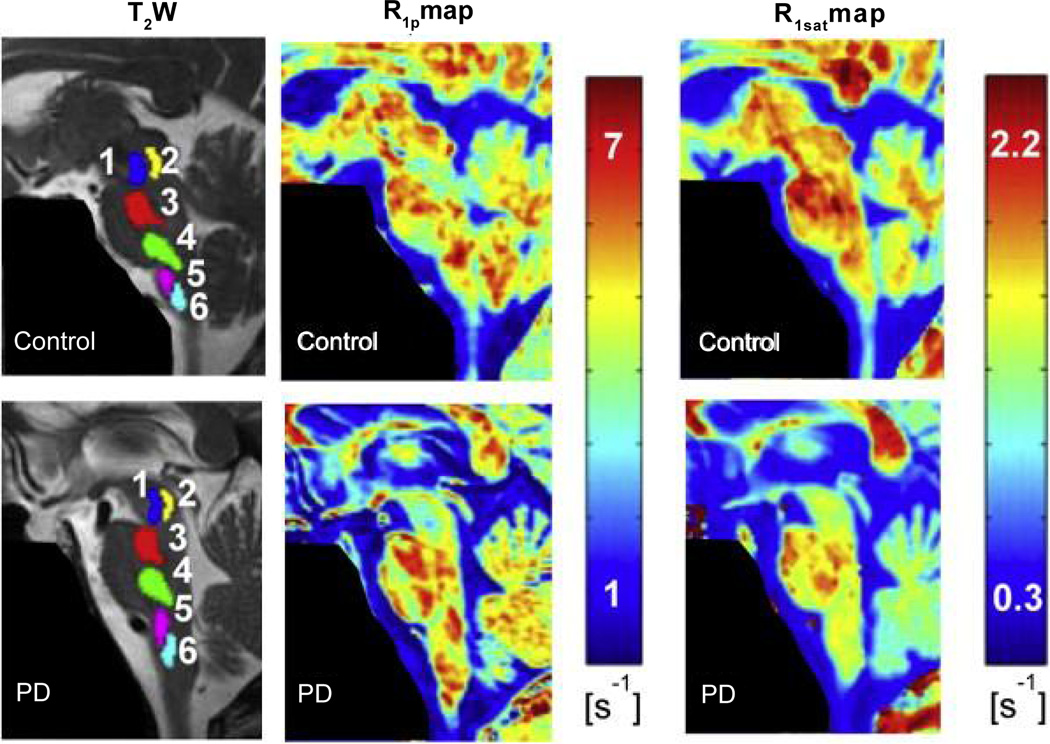
Rotating frame R_1_ρ maps (middle column), MT rate maps (R_1sat_) (right column), with relative T_2_-weighted (T_2_w) images (left column) from representative control subject (top row) and PD patient (bottom row). Regions of interest (ROIs) – as depicted on T_2_w images – were obtained in maps from six areas: (1) medial raphe nucleus; (2) dorsal raphe nucleus; (3) nucleus raphe pontis; (4) nucleus raphe magnus; (5) nucleus raphe pallidus; (6) nucleus raphe obscuris [23].

Rotating frame relaxation constants can be measured during the application of adiabatic pulses [24]. In this case, the adiabatic R_1ρ_(t) and R_2ρ_(t) are time-dependent longitudinal and transverse relaxation rate constants respectively, which characterize decay of magnetization during application of RF pulses operating in the adiabatic regime. As a result, adiabatic R_1ρ_ (≡1/ T_1ρ_) and R_2ρ_ (≡1/ T_2ρ_) provide novel tissue MRI contrast. The R_1ρ_ and R_2ρ_ measured during adiabatic pulses were demonstrated to be sensitive to neural integrity and iron accumulation, respectively [8, 9, 25, 26]. In a validation study of a *aphakia* mouse model, T_1ρ_ separated *aphakia* versus wild-type mice in the substantia nigra compacta (SNc) where there is a congenital absence of dopaminergic neurons [25]. In studies of PD patients and healthy controls, adiabatic methods are able to detect midbrain changes in PD [8, 9]. Additional work has shown that adiabatic methods demonstrate midbrain and medullary changes in PD as compared to controls [23]. In figure 3, the representative R_1ρ_ and R_1sat_ maps from control (top) and PD (bottom) subjects are shown. The differences between the R_1ρ_ values measured from a rostral region used as internal control per each subject (here identified by region of interest, ROI-1) minus the R_1ρ_ values measured from medullary nuclei (i.e., ROI-5 and ROI-1 vs. ROI-6) were altered in patients relative to control subjects (p=0.004 and p=0.033, respectively). Differences in R_1ρ_ values were 6 and 8 times larger in patients than in controls when comparing ROI-1 vs. ROI-5 and ROI-1 vs. ROI-6, respectively. Since R_1ρ_ values in ROI-1 were not different between patients and controls (p=0.25), these findings represent a change in imaging parameters from areas that contain medullary nuclei that are known to be affected in PD. Interestingly, no statistical differences were observed between patients and controls when considering R_1sat_. This was attributed to differential sensitivity to the exchange regime between T_1ρ_ and T_1sat_ [22]. Together, the findings of this study might indicate changes in fundamental tissue MR parameters that occur prior to neuronal death within the medullary nuclei.

## Relaxations along a Fictitious Field (RAFF)

A potential limitation to the widespread exploitation of rotating frame relaxation in PD is the required RF power delivered to the sample (i.e., specific absorption rate-SAR), which can result in tissue heating. However, RF power can sometimes be reduced by using off-resonance irradiation to create the locking field, B_eff_ [27, 28]. Recently colleagues at the Center for Magnetic Resonance Research (CMRR) have developed a novel rotating frame relaxation experiment called Relaxation along a Fictitious Field (RAFF), which comprises T_1ρ_ and T_2ρ_ mechanisms by exploiting relaxation in a second rotating frame. RAFF was able to provide a greater contrast in tissues of the SN as compared to T_1ρ_ and T_2ρ_and specifically it was better than all other methods in separating the SN into its various subregions, i.e. the pars compacta from pars reticulata [29]. Additional studies are warranted to sort out its utility.

## Diffusion Tensor Imaging (DTI)

Diffusion tensor imaging (DTI) provides structural data based on directionally restrained diffusion of water (anisotropy) within fiber tracts. Pathology disturbs the natural state of anisotropy and this can be exploited with DTI imaging. Specifically, the loss of restriction of water movement within damaged fiber bundles results in reduced anisotropy, which is characterized as a reduction in fractional anisotropy (FA). One group has shown changes in mean diffusivity in a cohort of individuals with RBD, a possible precursor to PD [30]. DTI has its limitations in determining directional and spatial anisotropy; hence some researchers have used probabilistic and streamline tractography that address these challenges.

## Resting-state MRI

The focus of resting-state MRI is on brain activity that occurs in the absence of externally triggered activity. Even in a “resting state” there are physiological variations in brain activity and accompanying blood flow alterations that manifest as fluctuations in the MRI blood oxygen level dependent (BOLD) signal. Spontaneous correlations in BOLD signal can be utilized to determine the “functional connectivity” between different regions. There have been a number of studies in PD that have shown alterations in sensorimotor circuitry and integration that accompanies motor and non-motor symptoms [31–36]. Measurement of fluctuation can be done using methods such as the amplitude of low frequency fluctuation or ALFF to assess for an index of resting-state brain activity based on the blood flow variability [35]. Resting-state methods allow for the determination of spontaneously occurring brain networks, which may distinguish PD from controls; however, in one study 1/3 of those with PD and 1/5 of controls had unusable data due to motion artifact, which may be partially due for the need to assess subjects when they had been off medications for at least 12 hours [35]. Hence while resting-state fMRI methods are able to provide a rapid and whole brain view of PD additional studies are needed to determine its role in understanding clinical subtypes and features of PD.

## *In vivo* Magnetic Resonance Spectroscopy (MRS)

**Figure 4:**
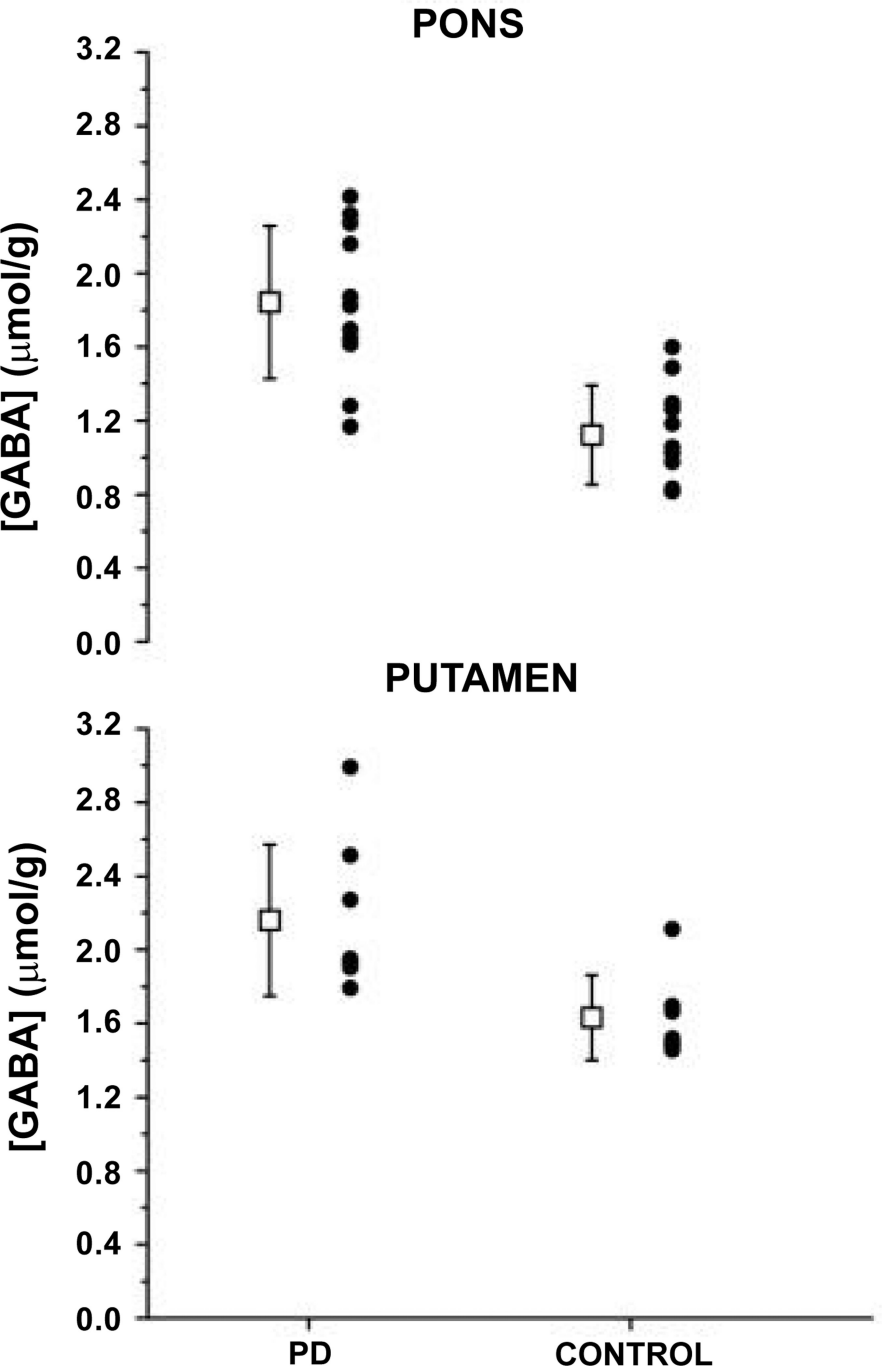
GABA concentrations in pons and putamen in individuals with Parkinson’s disease (PD) and controls; together with means (boxes) and standard deviations (error bars) [37].

MRS has been limited due to low sensitivity of methods and the low concentrations of metabolites of interest. High field MRS (Figure 4) with its greater sensitivity has overcome some limitations as shown by Emir et al. who demonstrated the ability to measure absolute concentrations of neurochemicals within the substantia nigra and other brainstem regions [37].

Meanwhile, MRS imaging (MRSI) can measure cerebral metabolic rates of oxygen (CMRO_2_) and ATP (CMR_ATP_) and to correlate neuroenergetics with specific brain functions. CMRO_2_ measurements are achieved using inhaled ^17^O_2_ gas which is ultimately incorporated into labeled water (H_2_^17^O) in brain tissue, which is detectable by in vivo ^17^O MRS [38, 39]. This method allows the determination of the role of oxygen metabolism in normal brain function and disease to complement functional MRI studies that utilize the BOLD contrast and are sensitive to cerebral blood flow.

Another important development includes *in vivo* ^31^P MRSI which generates measurements of intracellular pH, metabolites of ATP, ADP and phosphocreatine (PCr), among others [39]. The combination of MRSI and magnetization transfer imaging allows for the measurement of ATP metabolic rate (CMR_ATP_), and hence oxidative phosphorylation, a measure of cerebral mitochondrial function. This may prove useful in PD, in which mitochondrial dysfunction is thought to play a key role.

## Detection of Structural Changes

Starting from in the early 1990s, researchers have attempted at evaluating structural changes in brain regions critical to PD as revealed by various MRI anatomical methods. For instance, based on T_2_-weighted images, nigral ROIs of PD were compared with those of control subjects, and reduction of the size of nigral regions was observed in PD patients [40]. More recently “un-biased” methods such as voxel-based morphometry (VBM) have been used that don’t focus on a specific ROI. With VBM there is standardization of data and then voxel-by-voxel comparison between group data to evaluate for differences in signal intensity. VBM methods usually utilize 1.5 or 3T T_1_ anatomical data, which may not be sufficiently sensitive to detect structural changes in PD until there is substantial disease progression and the presence of accompanying dementia [41, 42]. However, one group has shown that VBM may be able to detect brainstem changes in idiopathic rapid eye movement sleep behavioral (iRBD) – suggesting its use early in the disease process as RBD may represent a precursor to PD [43].

## Clinical Applications

MRI methods are making their way into the clinic by aiding the neurosurgeon in planning deep brain stimulation (DBS) surgery [43]. Secondly, multi-modality approaches may increase sensitivity to disease states, as shown for example in the combination of structural and iron sensitive imaging [44, 45]. It is hoped that cross-sectional and longitudinal studies will provide insights about the ability of such methods to provide a correlate to disease severity and progression.

## References

[1] Tanner CM, Goldman SM (1996). Epidemiology of Parkinson’s disease. Neurol Clin.

[2] Rutledge JN, Hilal SK, Silver AJ, Defendini R, Fahn S (1987). Study of movement disorders and brain iron by MR. AJR Am J Roentgenol.

[3] Gorell JM, Ordidge RJ, Brown GG, Deniau JC, Buderer NM (1995). Increased iron-related MRI contrast in the substantia nigra in Parkinson’s disease. Neurology.

[4] Cho ZH, Oh SH, Kim JM, Park SY, Kwon DH (2011). Direct visualization of Parkinson’s disease by in vivo human brain imaging using 7.0T magnetic resonance imaging. Mov Disord.

[5] Zecca L, Youdim MB, Riederer P, Connor JR, Crichton RR (2004). Iron, brain ageing and neurodegenerative disorders. Nat Rev Neurosci.

[6] Zecca L, Wilms H, Geick S, Claasen JH, Brandenburg LO (2008). Human neuromelanin induces neuroinflammation and neurodegeneration in the rat substantia nigra: implications for Parkinson’s disease. Acta Neuropathol.

[7] Jin L, Wang J, Zhao L, Jin H, Fei G (2011). Decreased serum ceruloplasmin levels characteristically aggravate nigral iron deposition in Parkinson’s disease. Brain.

[8] Michaeli S, Oz G, Sorce DJ, Garwood M, Ugurbil K (2007). Assessment of brain iron and neuronal integrity in patients with Parkinson’s disease using novel MRI contrasts. Mov Disord.

[9] Nestrasil I, Michaeli S, Liimatainen T, Rydeen CE, Kotz CM (2010). T1rho and T2rho MRI in the evaluation of Parkinson’s disease. J Neurol.

[10] Wang Y, Butros SR, Shuai X, Dai Y, Chen C (2012). Different iron-deposition patterns of multiple system atrophy with predominant parkinsonism and idiopathetic Parkinson diseases demonstrated by phase-corrected susceptibility-weighted imaging. AJNR Am J Neuroradiol.

[11] Manova ES, Habib CA, Boikov AS, Ayaz M, Khan A (2009). Characterizing the mesencephalon using susceptibility-weighted imaging. AJNR Am J Neuroradiol.

[12] Gupta D, Saini J, Kesavadas C, Sarma PS, Kishore A (2010). Utility of susceptibility-weighted MRI in differentiating Parkinson’s disease and atypical parkinsonism. Neuroradiology.

[13] Lenglet C, Abosch A, Yacoub E, De Martino F, Sapiro G (2012). Comprehensive in vivo mapping of the human basal ganglia and thalamic connectome in individuals using 7T MRI. PLoS One.

[14] Abosch A, Yacoub E, Ugurbil K, Harel N (2010). An assessment of current brain targets for deep brain stimulation surgery with susceptibility-weighted imaging at 7 tesla. Neurosurgery.

[15] Haacke EM, Mittal S, Wu Z, Neelavalli J, Cheng YC (2009). Susceptibility-weighted imaging: technical aspects and clinical applications, part 1. Am J Neuroradiol.

[16] Balaban RS, Ceckler TL (1992). Magnetization transfer contrast in magnetic resonance imaging. Magn Reson Q.

[17] Tambasco N, Belcastro V, Sarchielli P, Floridi P, Pierguidi L (2011). A magnetization transfer study of mild and advanced Parkinson’s disease. Eur J Neurol.

[18] Anik Y, Iseri P, Demirci A, Komsuoglu S, Inan N (2007). Magnetization transfer ratio in early period of Parkinson disease. Acad Radiol.

[19] Eckert T, Sailer M, Kaufmann J, Schrader C, Peschel T (2004). Differentiation of idiopathic Parkinson’s disease, multiple system atrophy, progressive supranuclear palsy, and healthy controls using magnetization transfer imaging. NeuroImage.

[20] Morgen K, Sammer G, Weber L, Aslan B, Muller C (2011). Structural brain abnormalities in patients with Parkinson disease: a comparative voxel-based analysis using T1-weighted MR imaging and magnetization transfer imaging. AJNR Am J Neuroradiol.

[21] Tambasco N, Pelliccioli GP, Chiarini P, Montanari GE, Leone F (2003). Magnetization transfer changes of grey and white matter in Parkinson’s disease. Neuroradiology.

[22] Mangia S, De Martino F, Liimatainen T, Garwood M, Michaeli S (2011). Magnetization transfer using inversion recovery during off-resonance irradiation. Magn Reson Imaging.

[23] Tuite PJ, Mangia S, Tyan AE, Lee MK, Garwood M (2012). Magnetization transfer and adiabatic R 1ρ MRI in the brainstem of Parkinson’s disease. Parkinsonism Relat Disord.

[24] Michaeli S, Sorce D, Garwood M (2008). T-2 rho and T-1 rho adiabatic relaxations and contrasts. Curr Anal Chem.

[25] Michaeli S, Burns TC, Kudishevich E, Harel N, Hanson T (2009). Detection of neuronal loss using T(1rho) MRI assessment of (1)H(2)O spin dynamics in the aphakia mouse. J Neurosci Methods.

[26] Mitsumori F, Watanabe H, Takaya N (2009). Estimation of brain iron concentration in vivo using a linear relationship between regional iron and apparent transverse relaxation rate of the tissue water at 4.7T. Magn Reson Med.

[27] Bendall MR, Garwood M, Uqurbil K, Pegg DT (1987). Adiabatic refocusing pulse which compensates for variable rf power and off-resonance effects. Magn Reson Med.

[28] Bendall MR, Pegg DT (1986). Uniform sample excitation with surface coils for in vivo spectroscopy by adiabatic rapid half passage. J Magn Reson.

[29] Mangia S, Traaseth NJ, Veglia G, Garwood M, Michaeli S (2010). Probing slow protein dynamics by adiabatic R(1rho) and R(2rho) NMR experiments. J Am Chem Soc.

[30] Scherfler C, Frauscher B, Schocke M, Iranzo A, Gschliesser V (2011). White and gray matter abnormalities in idiopathic rapid eye movement sleep behavior disorder: a diffusion-tensor imaging and voxel-based morphometry study. Ann Neurol.

[31] Wu T, Wang L, Hallett M, Chen Y, Li K (2011). Effective connectivity of brain networks during self-initiated movement in Parkinson’s disease. Neuroimage.

[32] Wu T, Long X, Zang Y, Wang L, Hallett M (2009). Regional homogeneity changes in patients with Parkinson’s disease. Hum Brain Mapp.

[33] Kwak Y, Peltier S, Bohnen NI, Müller ML, Dayalu P (2010). Altered resting state cortico-striatal connectivity in mild to moderate stage Parkinson’s disease. Front Syst Neurosci.

[34] Baudrexel S, Witte T, Seifried C, von Wegner F, Beissner F (2011). Resting state fMRI reveals increased subthalamic nucleus-motor cortex connectivity in Parkinson’s disease. Neuroimage.

[35] Skidmore FM, Yang M, Baxter L, von Deneen KM, Collingwood J (2011). Reliability analysis of the resting state can sensitively and specifically identify the presence of Parkinson disease. Neuroimage.

[36] Krajcovicova L, Mikl M, Marecek R, Rektorova I (2012). The default mode network integrity in patients with Parkinson’s disease is levodopa equivalent dose-dependent. J Neural Transm.

[37] Emir UE, Tuite PJ, Öz G (2012). Elevated pontine and putamenal GABA levels in mild-moderate Parkinson disease detected by 7 tesla proton MRS. PLoS One.

[38] Zhu XH, Chen W (2011). In vivo oxygen-17 NMR for imaging brain oxygen metabolism at high field. Prog Nucl Magn Reson Spectrosc.

[39] Zhu XH, Du F, Zhang N, Zhang Y, Lei H (2009). Advanced In Vivo Heteronuclear MRS Approaches for Studying Brain Bioenergetics Driven by Mitochondria. Methods Mol Biol.

[40] Pujol J, Junqué C, Vendrell P, Grau JM, Capdevila A (1992). Reduction of the substantia nigra width and motor decline in aging and Parkinson’s disease. Arch Neurol.

[41] Price S, Paviour D, Scahill R, Stevens J, Rossor M (2004). Voxel-based morphometry detects patterns of atrophy that help differentiate progressive supranuclear palsy and Parkinson’s disease. Neuroimage.

[42] Focke NK, Helms G, Scheewe S, Pantel PM, Bachmann CG (2011). Individual voxel-based subtype prediction can differentiate progressive supranuclear palsy from idiopathic Parkinson syndrome and healthy controls. Hum Brain Mapp.

[43] Sadikot AF, Chakravarty MM, Bertrand G, Rymar VV, Al-Subaie F (2011). Creation of Computerized 3D MRI-Integrated Atlases of the Human Basal Ganglia and Thalamus. Front Syst Neurosci.

[44] Péran P, Cherubini A, Assogna F, Piras F, Quattrocchi C (2010). Magnetic resonance imaging markers of Parkinson’s disease nigrostriatal signature. Brain.

[45] Du G, Lewis MM, Styner M, Shaffer ML, Sen S (2011). Combined R2* and diffusion tensor imaging changes in the substantia nigra in Parkinson’s disease. Mov Disord.

